# Pre-Harvest Treatment of Chitosan Oligosaccharides Improved Strawberry Fruit Quality

**DOI:** 10.3390/ijms19082194

**Published:** 2018-07-27

**Authors:** Yanqiu He, Santosh Kumar Bose, Wenxia Wang, Xiaochen Jia, Hang Lu, Heng Yin

**Affiliations:** 1 Dalian Institute of Chemical Physics, Chinese Academy of Sciences, Dalian 116023, China; yanqiuhe.dicp@gmail.com (Y.H.); santosh@dicp.ac.cn (S.K.B.); wangwx@dicp.ac.cn (W.W.); jiaxiaochen@dicp.ac.cn (X.J.); 2 Dalian Ocean University, 52# Heishijiao Street, Shahekou District, Dalian 116023, China

**Keywords:** chitosan oligosaccharide, strawberry, pre-harvest, quality, antioxidant, gene expression

## Abstract

Chitosan oligosaccharide (COS), derived through hydrolysis of chitosan, has been proved to be an effective plant immunity elicitor, eco-friendly, and easily soluble in water, and influenced several secondary metabolites content to improve fruit qualities. COS are widely used in agriculture to improve the defense response in plants. The purpose of this study was to investigate the pre-harvest treatment effect of COS on the quality of strawberry (*Fragaria* × *ananassa cv.* *qingxiang*). COS was dissolved in distilled water at a concentration of 50 mg·L^−1^ and sprayed at four different growth stages of strawberry plants, namely seedling stage, before flowering, fruit coloring (the stage of fruit from white to red) and full bloom. Uniform size, shape, color, without any visible damage, and disease-free fruits were harvested for determining the quality. The results showed that the fruit firmness, viscosity, lignin, sugars, protein, total soluble solid, and titratable acidity content increased in COS-treated fruits compared to control. In addition, COS pre-harvest treatment had a positive effect on anthocyanin, total phenol, flavonoid, vitamin C content and DPPH(2,2-diphenyl-1-picrylhydrazyl) scavenging activity of strawberry. Moreover, COS also increased the cell wall composition and regulated gene expression of some important enzymes involved in ethylene compound biosynthesis and cell wall degradation. The finding of this study suggests that pre-harvest application of COS is very useful for improving quality and antioxidant capacity of strawberry.

## 1. Introduction

Strawberry (*Fragaria* × *ananassa cv. qingxiang*) is one of the most popular and widely consumed berries due to its taste, sweetness and healthy function. The taste of strawberry is related to its hardness, viscosity, sugars, protein, total soluble solid, and titratable acidity content [1]. It is a good source of polyphenolic compounds such as flavanols and has antioxidant activity [2]. This, together with higher vitamin C content in strawberries, contributes beneficial effects on the maintenance of consumer health [3]. The strawberry has higher antioxidant activities than orange, grape, banana, apple, etc. [4].

Pesticides are widely applied in most strawberry farmland during growth and development. High pesticide residues are reported in the fruits, which are harmful for consumer’s health. Therefore, nowadays, consumers demand more natural, environmentally friendly fruit production, with high quality and without any chemical preservatives and pesticide residues [5]. To decrease the pesticide residues and to enhance the nutritional quality of fruits and vegetables, an effective eco-friendly compound is essential. 

Some reports noticed that the application of chemical agents to enhance fruit quality and shelf life increase the risk of adding contaminants with food products and results in negative environmental impact. Another eco-friendly natural compound, chitosan, has the potential to increase shelf life and fruit quality, but low solubility of bulk chitosan in aqueous media limits its wide spectrum of application.

Chitosan oligosaccharide (COS), derived through hydrolysis of chitosan [6], is a water-soluble compound and has been proved as an effective elicitor of plant immunity. Generally, elicitor pretreatment is an advanced method for plant resistance because it costs less energy than directly induced defense and is less harmful to plant growth [7]. In another experiment, COS significantly reduced the disease lesion size on tomato fruits when the tomato plants were pretreated with 1.0% or 2.5% (*w*/*v*) COS solution 10 days before being inoculated with *Colletotrichum* sp. [8]. COS has been considered a potent plant immunity elicitor and used on different plants such as *Arabidopsis*, tobacco and grapevine [9,10]. The number of spikes, grains per spike and total grain yield were increased by spraying COS at tillering stage of wheat [11]. However, another reporter [12] noticed that chitosan is a teratogenic compound when a high dose of chitosan (150 mg/kg body weight) is administrated orally to Wistar female rats. In our research, we used a very low dose, 50 mg/L, as foliar spray. Furthermore, COS is easily soluble in water and it is not harmful, which also supports many previous reports. COS have been the subject of increased attention in terms of their pharmaceutical and medicinal applications, due to their nontoxic and high-solubility properties as well as their positive physiological effects [13]. Gol et al. [14] also reported that plant elicitors such as chitosan, abscisic acid, methyl jasmonate and some other chemical agents have been reported to promote the quality and antioxidant activity of strawberry when exogenously applied. COS treatment promoted polyphenol content in Greek Oregano [15] and improve vitamin and polyphenol content in cherries [16]. These results suggest that COS not only triggers plant immunity, but also influences several secondary metabolites in plant to improve quality of fruits. COS are considered to be an environmentally friendly plant regulation substance, owing to its high solubility, non-toxicity and biocompatibility. Therefore, it is of interest to study the effect of COS on strawberry fruit quality. 

Research on the role of ethylene in fruit ripening and its signaling transduction pathways have been studied extensively in recent decades. Previous reports indicate that ethylene is possibly involved in the regulation of nonclimacteric fruit ripening [17]. The role of ethylene in climacteric fruit ripening and its molecular mechanisms are well studied but in respect of nonclimacteric fruits is far behind. Formation of 1-aminocyclopropane-1-carboxylic acid (ACC) from S-Adenosyl methionine (SAM) via ACC synthase (ACS) activity, and conversion of ACC to ethylene through ACC oxidase (ACO). Fruit softening is associated with cell wall disassembly and during fruit softening, pectin and hemicellulose in cell walls undergo solubilization and depolymerization, which contributes to cell wall loosening [18]. Most of the studies on ripening-related cell wall hydrolyses have examined the activities of pectin esterase (PE), pectin lyase (PL), polygalacturonase (PG) and endo-1,4-d-glucanase (EG) in various fruit [19]. However, to our knowledge, there is no scientific literature available regarding the effect of COS pre-harvest treatment on quality characteristics of strawberry fruit. Therefore, the present study has been undertaken with the objective of elucidating the potential of COS on quality improvement of strawberry.

## 2. Results and Discussion

### 2.1. Effect of COS Pre-Harvest Treatment on Strawberry Fruit Texture

**Figure 1:**
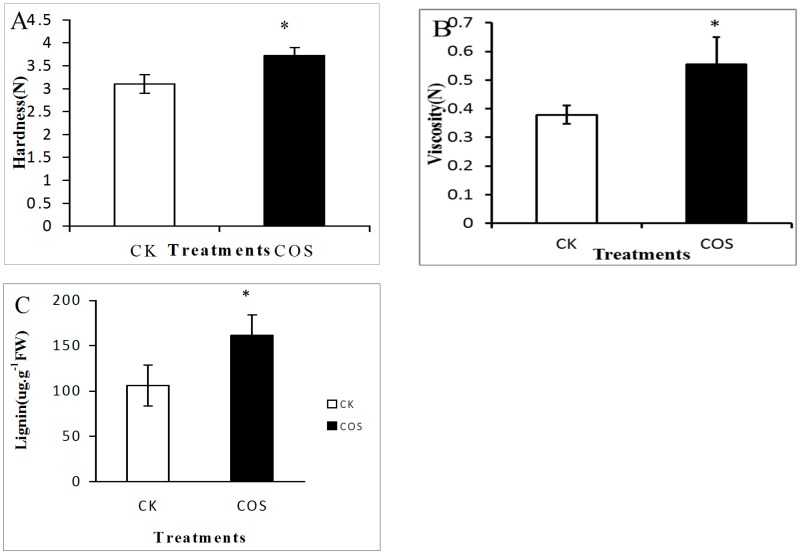
Effect of COS pre-harvest treatment on hardness (**A**); viscosity (**B**) and lignin (**C**) content of strawberry fruit. Data represent the means ± SD. The * represent means significantly different according to *T* test at *p* < 0.05 level. CK: Spraying water pre-harvest; COS: Spraying COS pre-harvest.

Hardness is an important physical parameter used to assess the quality of fruits during ripening. At harvest, strawberry fruits showed different hardness values that could be due to different lignin content. Hardness, viscosity and lignin content of COS-pre-treated strawberries were significantly higher (*p* < 0.05) than untreated fruits. The hardness of COS-pre-treated fruits was 3.73 N, while in untreated fruits was 3.10 N (Figure 1A). The viscosity also increased in COS-treated group (Figure 1B).

The lignin content of COS-treated fruit was approximately 50% higher than untreated fruit (Figure 1C). Hardness and viscosity are very important indices of fruit texture. The increased hardness in fruits may be due to the increased viscosity and the strength of cell-to-cell bonding [13]. Yan et al. [20] reported that the COS pre-harvest treatment increased the hardness in jujube fruits. In most cases, the viscosity of strawberry is closely related to the content of sugars, which are the composition of the cell wall. Constant increasing of lignin content in COS-treated fruits indicated that COS could induce lignin biosynthesis. The accumulation of lignin in the cell walls of higher plants provides hardness and the stability of the structure [21].

### 2.2. Effect of COS Pre-Harvest Treatment on Strawberry Cell Wall Components

The hardness of COS-treated fruit was higher than the untreated fruits, and in general, the difference in hardness was reflected by the differences in components of the cell wall. Five major compositions of cell wall were extracted in this study. Except for water-soluble pectin, the other four cell wall components were significantly enhanced by COS pre-harvest treatment compared to untreated fruits (Table 1). The crude cell wall content of control and COS-treated fruits were 1.8% and 2.2%, respectively. The strawberry fruit softening has been characterized by the solubilization and depolymerization of pectins from cell walls [22]. According to previous report, the ionic pectin, its covalent bonding, hemicellulose, and cellulose content were closely related to the texture [21]. They also reported that during the ripening process, only the ionic cross-links remain and so the hardness of fruits is partly due to the content of ionic bond pectin [19]. The increase of hemicelluloses and celluloses are closely correlated with the hardness. The cellulose and pectin may be locked together by covalent links between some xyloglucan molecules and pectin [23]. Our results suggested that COS pre-harvest treatment increased the proportion of the ionic pectin, its covalent bonding, hemicellulose and cellulose content. These changes in the cell wall composition might have played a role in the increased hardness of strawberry.

### 2.3. Effect of COS Pre-Harvest Treatment on Strawberry Quality and Taste

Quality and taste are important for the value of fruits. Soluble solids content (SSC), titratable acidity (TA), total sugar (TS) and the ratio of TS/TA are important factors for evaluating fruit quality [24].

The pre-harvest application of COS increased the sugar content (TS) of strawberry which improved the taste. Previous reports showed that pre-harvest spraying of antagonistic yeast plus chitosan increased the total soluble solid (TSS) content in grape [25].

**Figure 2:**
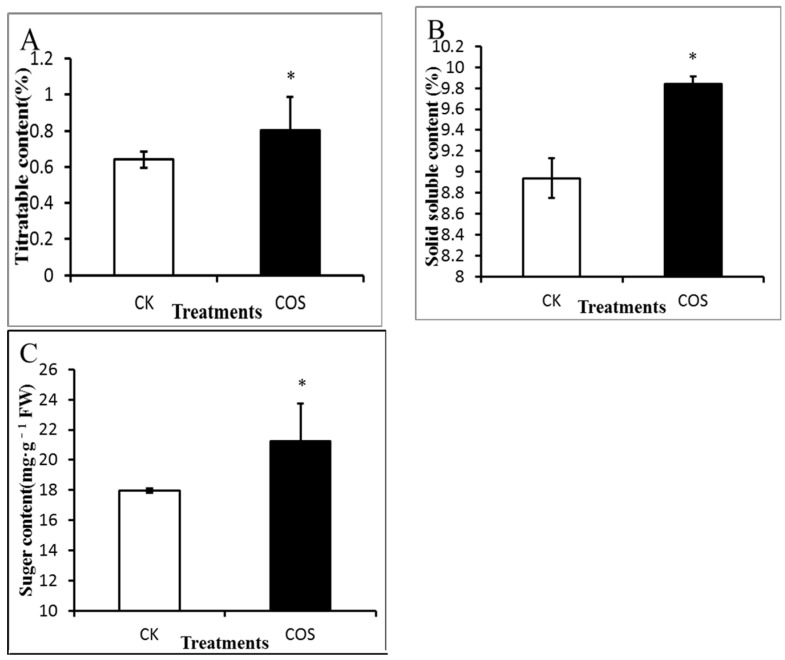
Effect of COS pre-harvest treatment on quality of strawberry fruit. (**A**) Titratable acidity; (**B**) Solid soluble content; (**C**) Sugar content. Data represent the means ± SD. The symbol star (*) means significantly different according to *T* test at *p* ≤ 0.05 level. CK: Spraying water pre-harvest; COS: Spraying COS pre-harvest.

The TA content significantly increased (*p* < 0.05) in the COS-treated fruit compared to the control (Figure 2A). SSC content in the control fruit was 8.94%, whereas it was 9.84% in COS-treated fruits (Figure 2B). From the result it was noted that TS content in strawberries was significantly higher (*p* < 0.05) in COS-treated fruits (21.24 mg·g^−1^) than the control fruits (17.96 mg·g^−1^) (Figure 2C). Significant difference was also observed in the case of TS/TA ratio between COS (1.39) and control (1.04) group. The higher TS/TA ratio indicates better taste and quality of strawberry fruits in COS-treated group.

### 2.4. Effect of COS Pre-Harvest Treatment on Strawberry Antioxidant Activity

The DPPH assay is a common method to measure the antioxidant capacity of fruits.

**Figure 3:**
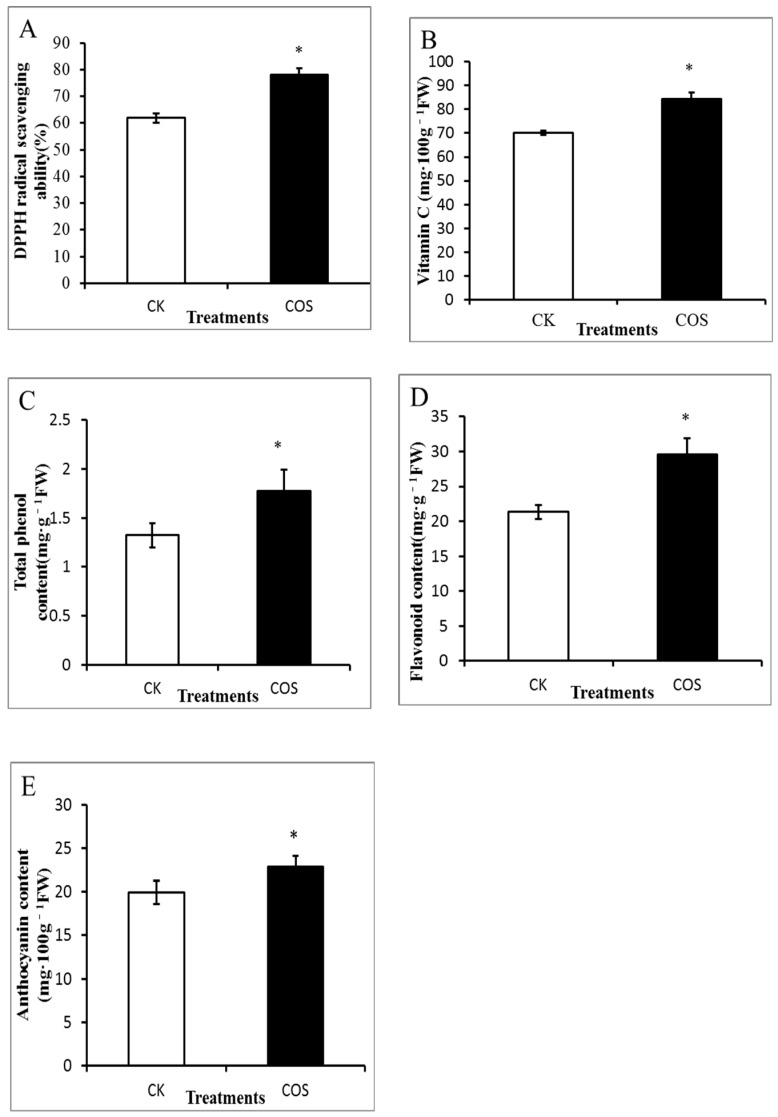
Effects of COS pre-harvest treatment on antioxidant activity of strawberry fruit. (**A**) DPPH scavenging activity; (**B**) Vitamin C content; (**C**) Total phenol content; (**D**) Flavonoid content; (**E**) Anthocyanin content. Data represent the means ± SD. The symbol star (*) means significantly different according to *T* test at *p* ≤ 0.05 level. CK: Spraying water pre-harvest; COS: Spraying COS pre-harvest.

In this study, it was found that the comparative DPPH free radical scavenging activity of the strawberry was significantly increased (*p* < 0.05) by pre-harvest spraying COS (16% higher than the control) (Figure 3A). The total antioxidant capacity of the strawberry depends mainly on the vitamin C [26], polyphenols, flavonoids and anthocyanins content [27]. Antioxidant activity based on free radical scavenging in strawberry fruit has a potential function in anti-hyperglycemia and anti-hypertension [28]. In our study, vitamin C, total phenol, flavonoids and anthocyanins content were increased, which implied that COS pre-harvest treatments have positive effects on antioxidant activity.

The vitamin C content of strawberry was significantly increased (*p* < 0.05) by pre-harvest spraying of COS and it was 20.02% higher than the control (Figure 3B). Vitamin C is a typical nutrient, and its content is often considered as a significant marker of overall nutrient quality [29]. Chitosan coatings improved the vitamin C content of strawberries; however, the bulk chitosan is poorly soluble in aqueous media which limits its wide application [14]. The phenolic compounds (a major secondary metabolite in plants) are well-known to have antioxidant properties [30]. The phenol content of the COS-treated group was significantly (*p* < 0.05) increased compared to the control group (Figure 3C). Calcium chloride treatment and temperature control methods have been used to promote the total phenol content in strawberry [31].

Flavonoids are a major group in the family of phenolic compounds with antioxidant and biological activity that have been identified in fruits [30]. As shown in Figure 3D, the flavonoid content of control and COS were 21.34 and 29.59 mg·g^−1^, respectively. The flavonoids from COS treatments increased 38% compared to control, which indicates that COS pre-harvest treatment significantly influenced the flavonoid content [32]. Flores and Ruiz del Castillo [33] reported that methyl jasmonate is a safe hormone and it could increase the flavonoid content of loquat fruit and red raspberry.

Anthocyanins are the most abundant flavonoid compounds in strawberry fruit. In this study, it was observed that pre-harvest spraying of COS significantly enhanced the accumulation of anthocyanins and it was higher in COS-treated fruits (23.63 mg·100 g^−1^) than the untreated fruits (21.11 mg·100 g^−1^) (Figure 3E). Total anthocyanin content of strawberry was increased by COS pre-harvest treatment, which may lead to higher antioxidant activity.

### 2.5. Effects of COS Pre-Harvest Treatment on Strawberry Gene Expression

**Figure 4:**
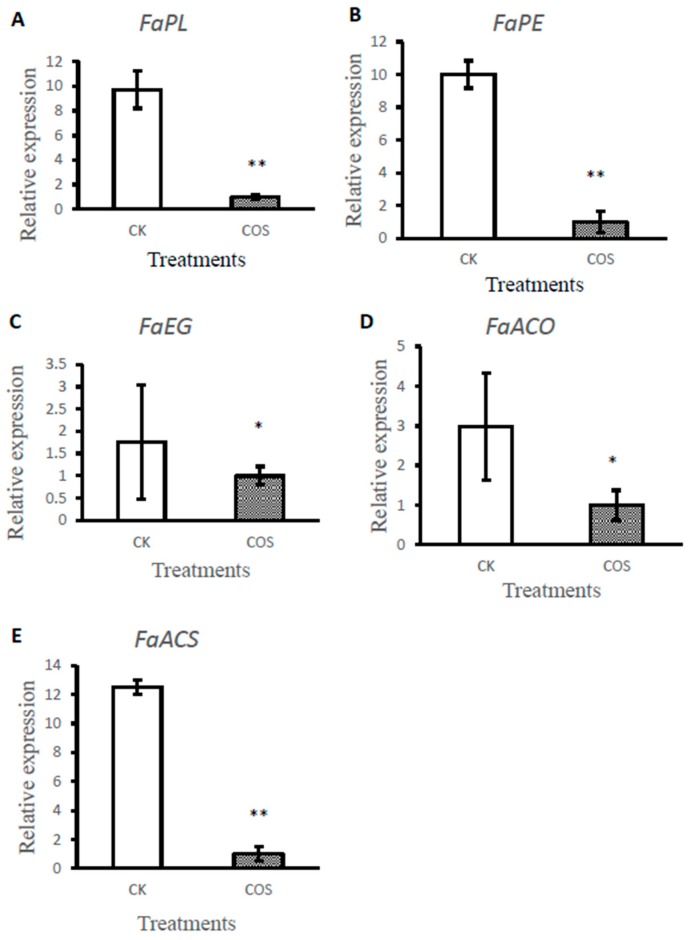
Effects of COS pre-harvest treatment on gene expression of some important enzymes involved in ethylene compound biosynthesis and cell wall degradation ((**A**) PL: Pectin lyase; (**B**) PE: Pectin esterase; (**C**) EG: Endoglucanase; (**D**) ACS: ACC synthetase; (**E**) ACO: ACC oxidase). The error bars represent the standard deviation of three biological replicates. Stars indicate significant differences. Data represent the means ± SD. The * are significantly different according to *T* test at *p* ≤ 0.05 level. The ** are significantly different according to *T* test at *p* ≤ 0.001 level. CK: Spraying water pre-harvest; COS: Spraying COS pre-harvest.

To explore the effects of COS on ethylene synthesis and cell wall degradation, the expression of important genes in these signaling pathways, including *FaACS*, *FaACO* (the ethylene synthesis pathway gene) *FaPL*, *FaPE*, and *FaEG* (the cell wall degradation pathway gene), were examined. *FaPL*, *FaPE* and *FaEG* have been reported to be involved in degradation of cell walls, resulting in tissue maceration [34] and the expressions of *FaPL*, *FaPE* and *FaEG* genes in fruits were associated with fruit softening**.** In this study, the expressions of *FaPL*, *FaPE* and *FaEG* genes were significantly suppressed by COS treatment compared to control, which indicates that COS-treated fruit were firmer than control. The result suggests that COS treatment could inhibit the gene expression of *FaPL*, *FaPE* and *FaEG* in strawberry, resulting in delayed degradation of the cell wall by inhibiting cell wall degradation enzyme gene expression, which is consistent with the results of cell wall fraction change. Previous reports suggested that the phytohormone ethylene plays an important role in strawberry fruit ripening [35]. Strawberry is a nonclimacteric fruit, but in recent years some studies reported that ethylene is possibly involved in the regulation of nonclimacteric fruit ripening. This study showed that COS influenced the ethylene pathway by reducing the expression of *FaACS* and *FaACO* gene. It was also observed that *FaACO* and *FaACS* genes expression were significantly suppressed by COS treatment compared to control fruits (Figure 4).

In this study, we focus on the quality improvement of strawberry by COS pretreatment. It is interesting that the fruit hardness is higher in the COS-treated group than in the control group. Besides the hardness, there is more crude cell wall content in the COS-treated group, especially the ionic pectin, hemicellulose and cellulose content, which are closely correlated with the hardness. These results implied that during post-harvest, the fruit of the COS-treated group may be easier to preserve. In addition, the gene expression analysis results also showed that the ripening-related gene is also in a relatively lower profiling expression, which also suggests that COS-treated fruit experience a slower post-harvest ripening process.

## 3. Materials and Methods

### 3.1. Chemicals

Chitosan oligosaccharide (degree of polymerization = 2~10; degree of deacetylation > 95%) was obtained from Dalian GlycoBio Co., Ltd., (Dalian, China). Folin-Ciocalteu reagent was procured from Solarbio (Beijing, China). trans-1,2-diaminocyclohexanetetraaceticacid (CDTA), 1,1-diphenyl-2-picrylhdrazyl (DPPH), gallic acid, vitamin C, sodium carbonate, sodium hydroxide, sulfuric acid and hydrochloric acid were purchased from Sigma (St. Louis, MO, USA). All other chemicals and solvents were of analytical grade and obtained from Chinese manufacturers.

### 3.2. Treatment of Strawberry

COS was dissolved in distilled water at a concentration of 50 mg·L^−1^. The COS solution was sprayed at the rate of 375 L·ha^−1^ in a commercial orchard (Zhuanghe City, China) at four different growth stages of strawberry plants, namely seedling stage, before flowering, fruit coloring (the stage of fruit from white to red) and full bloom. The control group was treated with equal amount of water at the same time.

Fresh and mature strawberry fruits were harvested (April 2015). Twenty fruits from each treatment group were selected, with uniform size, shape, color without any visible damage, disease for further study. For biological analyses the fruits were immediately frozen in liquid nitrogen, and stored at −80 °C.

### 3.3. Texture Analyses

“TA.XT.plus Texture Analyser” (Stable Micro Systems Ltd., Surrey, UK) along with the measuring probe P/5S (5 mm Spherical stainless steel, supplied with the Texture Analyser) were employed for texture determination. The system was equipped with texture profile analysis (TPA). Hardness was measured as the maximum penetration force (N) reached during tissue breakage. The maximum penetration force was set as 25 N. Other measurable parameters were: pretest speed 1 mms^−1^; test speed 1 mms^−1^ penetrating distance of 5 mm into the fruit. The measurement was triggered automatically at 0.01 N. The maximum force required for sample compression was calculated as an average of 10 measurements.

### 3.4. Preparation and Fractionation of Cell Wall

The cell wall preparation was done as described by Brummell et al. [19] with some modification. Five grams of frozen fruit material was homogenized in 20 mL of 80% ice-cold ethanol using a Polyton homogenizer. Insoluble residue was washed with 80% ice-cold ethanol, re-suspended in Tris-buffered phenol, precipitated with ethanol, washed with 95% ethanol, re-suspended in chloroform: methanol (1:1, *v*/*v*), and washed with acetone. Aliquots (100 mg) of acetone-insoluble cell wall fractions were sequentially extracted twice with CDTA for 24 h and Na_2_CO_3_ containing 0.1% NaBH_4_ for 24 h to isolate ionically and covalently bound pectin, respectively. The depectinated cell wall residue was stirred with 1 M KOH with the addition of 20 mM NaBH_4_ for 18 h at 20 °C; thereafter, it was filtered, and supernatant was collected as a hemicellulose (1 M KOH) fraction. The residue was purified by stirring it for 2 h with 4 M KOH containing 20 mM NaBH_4_. After filtration, samples were stirred three times with 0.1 M HCl at 85 °C for 30 min and then three times with 1 M NaOH at 85 °C, and finally rinsed with hot deionized water to obtain cellulose fraction.

### 3.5. Lignin Analysis

Lignin content of fruit was measured according to the method of Morrison [36] with some modifications. One gram strawberry was homogenized in ice-cold 95% ethanol using a plastic pestle and then using an ultrasonic disruptor. The homogenate was centrifuged at 3000× *g* for 10 min. The resultant pellet was washed three times with 95% ethanol and twice with a 1:2 (*v*/*v*) mixture of ethanol to hexane and allowed to air-dry at 37 °C overnight. The dried pellet was ultrasonically homogenized in acetic acid and centrifuged at 1000× *g* for 5 min. 300 mL 25% acetyl bromide in acetic acid was added to each sample. After heating at 70 °C for 30 min, 270 mL 2 M NaOH, 30 mL 7.5 M hydroxylamine hydrochloride, and 900 mL acetic acid were added to each sample. The samples were centrifuged at 1000× *g* for 5 min, and the absorbance of the supernatant was measured at 280 nm to determine the lignin content.

### 3.6. Total Sugars (TS) Analysis

The content of TS in ripe fruits was estimated by Anthrone-sulfuric acid method with minor modifications [37]. Five gram fruit was homogenized in 50 mL deionized water and the extract was boiled for 30 min. The sample was allowed to cool at room temperature, filtered and diluted with water to 100 mL. 1 mL of extract was mixed with 4 mL Anthrone-sulfuric acid reagent, boiled for 10 min and allowed to cool. The absorbance of the solution was measured at 620 nm and colorimeter. The standard curve was prepared by using pure glucose at a concentration of 0.1–1.0 mg/L in distilled water. Finally, the percentage of total sugar was determined by using the following formula:

$$\;\%\;\mathrm{Total}\;\mathrm{sugar}\;\left(\;g/100\;g\;\mathrm{of}\;\mathrm{fruits}\right)=\;\frac{\mathrm{Amount}\;\mathrm{of}\;\mathrm{sugar}\;\mathrm{obtained}}{\mathrm{Weight}\;\mathrm{of}\;\mathrm{samples}}\;\times 100\;$$

### 3.7. Soluble Solid Content (SSC) and Titratable Acidity (TA) Content

Ten strawberry fruits were selected randomly from control and COS treatment group and homogenized in a mortar, filtered through muslin cloth. The SSC content of the fruit juice was determined by measuring the refractive index with a hand-held refractometer (Shanghai optical instrument, 2 W) at 25 °C. The results were expressed as percentages [38].

TA was analyzed by titration method with 0.1 M NaOH to pH 8.3 using phenolphthalein as indicator and calculated using the following formula.

$$\begin{array}{l} \mathrm{Citric}\;\mathrm{acid}\;(\%)\\ =\frac{\mathrm{Titre}\;(\mathrm{mL})\;\times \;\mathrm{NaOH}\;\mathrm{normality}\;(0.1\;N)\;\times \;\mathrm{vol}.\;\mathrm{made}\;\mathrm{up}\;(50\;\mathrm{mL})\;\times \;\mathrm{citric}\;\mathrm{acid}\;\mathrm{eq}.\;\mathrm{weight}\;(64\;\mathrm{gm})\;\times \;100}{\mathrm{Vol}.\;\mathrm{of}\;\mathrm{sample}\;\mathrm{for}\;\mathrm{titrate}\;(5\;\mathrm{mL})\;\times \;\mathrm{wt}.\;\mathrm{of}\;\mathrm{sample}\;\mathrm{taken}\;(10\;g)\;\times \;1000} \end{array}\;$$

### 3.8. Antioxidant Activity

Fruits (5 g) were ground in liquid nitrogen with mortar and pestle. The powder was added to 75 mL of methanol containing 0.2% acetic in a conical flask and incubated for 2 h at 60 °C. After filtration, the residue was extracted with fresh solvent as described above. Filtrates were centrifuged at 10,000× *g* for 15 min and clear supernatant was stored at −20 °C for further analysis.

The antioxidant activity of strawberry was assessed according to the method described by Patras et al. [39]. Briefly, 2 mL of the extract was mixed with 2 mL of 0.1 mm DPPH solution and incubated at room temperature for 30 min in the dark. Absorbance was then read at 517 nm using methanol as a blank and ascorbic acid as a positive control. The radical scavenging effect was measured as a decrease in the absorbance of DPPH and calculated by using the following equation: scavenging activity (%) = (A_control_ − A_sample_)/A_control_ × 100.

### 3.9. Vitamin C Content

The Vitamin C content was estimated in accordance with the 2,6-dichlorophenolindophenol titration method [1]. Briefly, strawberry (5 g) was homogenized in 50 mL of 0.02 g·mL^−1^ oxalic acid solution and then centrifuged at 15,000× *g* for 15 min at 4 °C. The supernatant (10 mL) was titrated to a permanent pink color by 0.1% 2,6-dichlorophenolindophenol. Vitamin C concentration was calculated according to the titration volume of 2,6-dichlorophenolindophenol using following formula and expressed as mg·100 g^−1^ FW.

$$\;\mathrm{Ascorbic}\;\mathrm{acid}\;\left(\mathrm{mg}/100\;g\right)=\frac{\mathrm{Titre}\;\left(\mathrm{mL}\right)\times \mathrm{dye}\;\mathrm{factor}\;\times \mathrm{vol}.\mathrm{made}\;\mathrm{up}\;\left(\mathrm{mL}\right)\;\times 100}{\mathrm{Aliquot}\;\mathrm{used}\;\mathrm{for}\;\mathrm{estimation}\;\left(\mathrm{mL}\right)\;\times \mathrm{sampleweight}\;\left(g\right)}\;$$

### 3.10. Total Anthocyanins Content

Total anthocyanins were extracted by pH differential method [39]. Absorbance was measured at 510 nm and 700 nm in buffer at pH 1.00 and pH 4.5, using A = (A_510_ − A_700_) pH 1.0 − (A_510_ − A_700_) pH 4.5. Anthocyanin content was calculated by the following formula: Anthocyanins content (mg·100 g^−1^ FW) = [(A/€ × L) × V/m] × MW × 100. Where A is the absorbance, € the cyaniding-3-glucoside molar extinction coefficient (22,400), L the cell path length (1 cm), MW the molecular weight of anthocyanin (449.2).

### 3.11. Total Phenol Content (TPC)

Total phenol compounds were determined using the Folin-Ciocalteu method [40]. Briefly, for each test tube, 200 μL extract (matched control), 2 mL of 1:10 Folin-Ciocalteu’s reagent and 4 mL Na_2_CO_3_ (1 M) were added. The test tube was incubated for 2 h at room temperature in the dark and absorbance values were measured at 765 nm. Gallic acid (1 mg·mL^−1^) was used as the standard and the results were expressed as mg gallic acid eq. g^−1^ FW.

### 3.12. Flavonoids Content

The flavonoid content was estimated by a colorimetric assay [41]. One mL extract and 0.5 mL of 5% sodium nitrite was added in a 10 mL volumetric flask. After 6 min, 0.5 mL of 10% aluminum nitrate was added and allowed to stand for 6 min. Subsequently, 4% sodium hydroxide and 70% ethyl alcohol of 4 mL each was added and allowed to stand for 12 min. Finally, the absorbance was measured at 502 nm. Comparisons were made with standards of known rutin concentrations, and the results were expressed as mg·g^−1^ FW.

### 3.13. RNA Extraction, cDNA Preparation and Gene Expression Analysis

Frozen tissues (5 g) from at least 5 samples were ground in a mortar using a pestle in the presence of liquid nitrogen. Total RNA was extracted using the hot borate method as described Landi et al. [42]. RNA was quantified by absorbance at 260 nm. After the treatment with RNase-free DNase (Promega Biotech Ibérica. Madrid, Spain), RNA of the same quality was reverse transcribed into cDNA with AMV Reverse Transcriptase (Takara, Kusatsu, Japan). Dilutions of cDNA were used as templates in q-PCR total mRNA was reverse transcribed using polyT primers and the First Strand cDNA Synthesis Kit for RT-PCR (AMV) (Roche Farma, SA, Barcelona, Spain) following the manufacturer’s instructions.

The real time qPCR amplification was performed with gene-specific primers (Table 2). 26S18S gene (a housekeeping gene) was used as internal control for all the strawberry genes. Each reaction was performed in triplicate for each sample in 20 μL final volume containing 5 μL cDNA, 25 pmol specific primers, and 10 μL of Power SYBR Green PCR Master Mix (Applied Biosystems) according to the manufacturer’s protocol. PCRs were carried out using the ABIVeriti (Applied Biosystems, Foster City, CA, USA) for 2 min at 95 °C and then for 40 cycles as follows: 5 s at 95 °C, 10 s at 58 °C and 10 s at 72 °C. The specificity of the PCR amplification was confirmed with a melt curve analysis consisting of a 0.1 °C temperature gradient from 55 to 90 °C. Primer efficiencies were determined for each oligonucleotide pair. To account for different primer efficiencies, relative quantification of transcript levels to the reference. Relative expression levels were calculated using the 2^−^^ΔΔCt^ method [43].

### 3.14. Statistical Analyses

All the measurements were conducted at least triplicate. Data were expressed as mean ± SD (*n* = 3). The data were analyzed using the SPSS 19.0 (IBM, New York, NY, USA) software for analysis of variance and Duncan’s test. The significance was established at *p* ≤ 0.05.

## 4. Conclusions

The results of the present study indicate that the pre-harvest spraying of COS seemed to have a beneficial impact on quality improvement of strawberry. Fruit treated with COS had positive effect on fruit firmness, viscosity, lignin, vitamin C, total phenol and antioxidant activity. Moreover, COS also increased the cell wall content and suppressed the expression of genes involved in the ethylene signaling pathway, which reduced the softness and increased the shelf life of fruit. These results imply that COS may not only improve strawberry quality, but also have positive effect on extending its shelf life. Combined with its well-known plant defense induction activity, COS could be used as an eco-friendly, nontoxic substitute substance to traditional pesticides in fruit crops.

## Figures and Tables

**Table 1:** Effect of COS pre-harvest treatment on strawberry fruit cell wall composition.

Treatments	Fresh Weight (g)	Crude Cell Wall Extract					
		Total Quantity (g)	A (%)	B (%)	C (%)	D (%)	E (%)
CK	5	0.09	9.85 ± 3.05	6.34 ± 1.93	7.37 ± 1.40	24.42 ± 2.69	20.06 ± 2.26
COS	5	0.11^*^	8.30 ± 2.38	7.41 ± 2.52	9.88 ± 3.31 *	26.86 ± 1.78	24.24 ± 1.31 *

A: Water-soluble pectin; B: Ionic pectin; C: Covalent bonding type of pectin; D: Hemicellulose; E: Cellulose. Data represent the means ± SD. The symbol star (*) means significantly different according to *T* test at *p* ≤ 0.05 level. CK: Spraying water pre-harvest; COS: Spraying COS pre-harvest.

**Table 2:** Gene-specific oligonucleotides primers pairs used for RTqPCR. The accession number of each gene was obtained from GenBank.

Name Gene	Gene ID	Sequence of the 5–3 Primers, Forward/Reverse
*FaPL*	101301735	CTCGTTTGCGTATCGG TGCGTGCTCATTCCA
*FaPE*	101310153	TTGGACCACATTTCGC GGTCGGCTCATCTTTGT
*FaACO*	101298627	TACCTCAAGCACCTTCCTCGC TTAGTGCCAAAGGTAGGACTA
*FaACS*	AY912491	GAGAACACGAAACTCCAAG CCAAGAAGACATCAACCC
*FaEG*	101301481	AACGAGTTTGGTTGGGATAA GCAGGAACGATAGCGAAG
*26S-18S*	X58118	ACCGTTGATTCGCACAATTGGTCATCG TACTGCGGGTCGGCAATCGGACG

Note: The Ct values for each qRT-PCR reaction were normalized in relation to the Ct value corresponding to an interspacer 26S-18S strawberry RNA gene (housekeeping gene) using the primers Fa18S-U: 5′-ACCGTTGATTCGCACAATTGG TCATCG-3′ and Fa18S-L: 5′-TACTGCGGGTCGGCAATCGGACG-3′.
